# A very short version of the Visual Function Questionnaire (VFQ‐3oo7) for use as a routinely applied Patient‐Reported Outcome Measure

**DOI:** 10.1111/aos.14378

**Published:** 2020-03-18

**Authors:** Martijn S. Visser, Reinier Timman, Karlijn J. Nijmeijer, Hans G. Lemij, Emine Kilic, Jan J.V. Busschbach

**Affiliations:** ^1^ Section of Medical Psychology and Psychotherapy Department of Psychiatry Erasmus Medical Centre Rotterdam The Netherlands; ^2^ Rotterdam Ophthalmic Institute Rotterdam The Netherlands; ^3^ The Rotterdam Eye Hospital Rotterdam The Netherlands; ^4^ Department of Ophthalmology Erasmus Medical Centre Rotterdam The Netherlands

**Keywords:** patient reported outcome measure, item response theory, Rasch analysis, routine outcome monitoring, quality of life, ophthalmology

## Abstract

**Background:**

Patient‐reported outcome measures (PROMs) are valuable supplements in regular care to facilitate routine monitoring of quality of life from the patient’s perspective. The 25‐item National Eye Institute Visual Function Questionnaire (NEI‐VFQ‐25) is a widely used PROM in ophthalmology. However, the NEI‐VFQ‐25 is too time‐consuming and cumbersome for routine evaluations in regular care. The aim of this study is to construct a 7‐item questionnaire of which only 3 items are presented to the patient, by means of routing. This VFQ 3 out of 7 (VFQ‐3oo7) should have a minimal loss of information compared with the NEI‐VFQ‐25.

**Methods:**

An historical database including 3293 administrations of the NEI‐VFQ‐25 was constructed involving patients with retinal detachment, cataract, corneal diseases, glaucoma, macular degeneration, uveal melanoma and a normal population sample. The data were subjected to Rasch analyses, in particular a generalized partial credit model. Items were sorted on the latent trait and divided into seven categories. From each category, the item with the highest discriminative value was selected. Through routing, only three out of the seven remaining questions are used, where the answers navigate patients to a fitting trait level.

**Results:**

A one‐dimensional structure was considered fitting. The VFQ‐3oo7 showed a small loss of information compared with the total score of the NEI‐VFQ‐25: correlation 0.927 and a relative precision of 0.868.

**Conclusion:**

The very short, but valid, VFQ‐3oo7 can be applied to evaluate the patient's perceived vision‐related health status in routine evaluations of treatments in regular care, with a small burden for patients.

## Introduction

Patient‐reported outcome measures (PROMs) summarize the patients’ perceived functional ability, health and well‐being (Michelotti et al. 2017). Patient‐reported outcome measures (PROMs) in ophthalmology are considered a valuable supplement to medical outcomes in (cost)effectiveness evaluations in clinical trials and quality improvement at a population level (Somner et al. 2012; Denniston et al. 2014). Moreover, PROMs can be valuable in the consultation room in regular care to routinely monitor the patient’s perspective, consequently stimulating patient participation and shared clinical decision‐making (Boyce et al. 2014; Fung et al. 2016). With such routine measurements, PROMs are also helpful in making the quality of care more transparent to patients, the government and financing bodies such as insurers. However, systematic, routine measurement of PROMs does not take place in ophthalmology (Michelotti et al. 2017). For such routine use in regular care, measurement instruments should be short, practical and useful (Somner et al. 2012). Most of the available PROMs are valid and reliable for research, but not for such routine use (Somner et al. 2012; Michelotti et al. 2017).

When patients administer PROMs and other questionnaires on a routine basis to systematically provide data on the quality of treatments in terms of treatment outcome, this is referred to as routine outcome monitoring (ROM). For example, the National Health Service introduced in 2009 the routine use of PROMs for hip surgery, knee surgery, hernia repair and the treatment of varicose veins. Over 100 000 cases are administered each year (NHS 2018). The International Consortium for Health Outcome Measurement (ICHOM) provides another example, aiming to settle an international standard for routine administration of PROMs based on the framework developed at the Harvard Business School by Michael Porter (ICHOM 2018). In The Netherlands, healthcare providers and the ministry of health organized an online platform in 2017 for the benchmarking of PROMs (Zorgladder 2018). All patients administer a set of questionnaires at predetermined points during the therapy. In all these initiatives, the aim is to make the quality of treatments more insightful.

The National Eye Institute Visual Function Questionnaire (NEI‐VFQ‐25) is a widely used patient‐reported outcome measure (PROM) in clinical trials in Ophthalmology (Mangione et al. 2001). The NEI‐VFQ‐25 is applied in many eye disorders, such as cataract, age‐related macular degeneration, diabetic retinopathy, retinal detachment, corneal disease, uveal melanoma, glaucoma, retinitis and patients with low vision from any cause. However, the NEI‐VFQ‐25 with its current length of 39 items is too time‐consuming and cumbersome for patients for routine use in regular care. This could particularly be a burden for patients with eye disorders. First, problems with their eyesight may cause more difficulty in reading the questionnaires. Second, eye disorders present themselves mainly in elderly, who often suffer from a significant comorbidity. A high comorbidity may require more follow‐up visits and treatments, with more frequent PROMs as a consequence.

We targeted to reduce the administrative burden of the NEI‐VFQ‐25 and potentially prevent the phenomenon of respondent burden. Respondent burden occurs when respondents' motivation drops as a result of the length of a survey and the data quality begins to deteriorate. The aim of the current study was to construct a very short version, suitable for routine use in regular ophthalmic care with a minimal loss of information. So, the focus was to retain the range of the latent trait or traits as wide as possible while the scale will still be sensitive for patients with severe as well as with mild visual problems.

The current study is not the first attempt to shorten the NEI‐VFQ‐25, although our aims were more rigorous in reducing the number of items, our methods also differed from those in previous studies and the range of ophthalmic diseases is much broader. Fukuhara et al. (2013) constructed an 11‐item short version of the NEI‐VFQ‐25 using item response theory also referred to as Rasch analysis from glaucoma, cataract and macular degeneration data. They intended to retain information on all domains, and therefore included at least one item on each of these domains. In another study, by Kowalski et al. (2012), six items of the NEI‐VFQ‐25, without the additional 14 items, were selected, based on Rasch analysis from glaucoma and macular oedema data. The item selection was based on the goodness of fit of the items. In an iterative procedure, the ill‐fitting items were removed. Also, Kowalski et al. selected at least one item per domain. Rasch analysis makes the assumption of uni‐dimensionality. In 2010, Pesudovs reported issues concerning the NEI‐VFQ‐25 caused by multidimensionality in cataract patients (Pesudovs et al. 2010). He suggested using two domains: visual functioning and a socio‐emotional scale.

The operationalization of our aim was to create the ‘VFQ 3 out of 7’ (VFQ‐3oo7), by reducing the NEI‐VFQ‐25 to seven items. By using a smart routing, the patient would only have to answer 3 of the 7 items, as items out of the range would not be presented to the patient. For instance, if the first item already indicated that the patient had severe visual problems, items about minor problems would not be presented.

## Methods

The Medical Ethics Committee of the Erasmus Medical Centre (Rotterdam, the Netherlands) judged that according to Dutch law, this study did not require a formal approval, as the data were anonymized and had been collected in previously approved studies.

### Study sample

A sample of 2383 patients was collected from archival data pertaining to various eye disorders. Several of our data sources have been described before: corneal disease (van Cleynenbreugel et al. 2014), glaucoma (Islamaj et al. 2018), macular degeneration (Lushchyk et al. 2013), uveal melanoma (van Beek et al. 2018) and retinal detachment (de Jong et al. 2017). An exception was the cataract data and some of the macular degeneration data, which were both collected in the Rotterdam Ophthalmic Institute, but that had not been published before. The number of patients and the distribution of background variables published previously differed slightly from the data currently presented, because we used different inclusion and exclusion criteria. We only excluded patients that failed to fill in the questionnaire. In order to enhance the generalizability, we collected an additional sample of 910 people from the general population, stratified for age and gender. In total, we had 3293 administrations of the NEI‐VFQ‐25. The background variables are described in Table 1.

**Table 1:** Numbers and participant characteristics.

	Population							
	Retinal detachment*, *	Cataract*, *	Corneal diseases	Glaucoma	Macular degeneration*, *	Uveal melanoma	Normal	Total
Baseline	191	124	81	115	336	111	910	1868
Follow‐up 1	58		84	113	285	113		653
Follow‐up 2			24	105		110		239
Follow‐up 3			51	72		105		228
Follow‐up 4				51		80		131
Follow‐up 5				37		67		104
Follow‐up 6						62		62
Follow‐up 7						8		8
Total	249	124	240	493	621	656	910	3293
Gender								
Female (%)	53 (31)	68 (56)	52 (62)	55 (46)	212 (63)	54 (48)	456 (50)	950 (51)
Male (%)	118 (69)	53 (44)	32 (38)	65 (54)	125 (37)	59 (52)	454 (50)	906 (49)
Mean age ± sd	60.7 ± 13.3	69.9 ± 10.7	72.0 ± 8.3	59.9 ± 9.1	78.7 ± 8.5	60.4 ± 12.7	69.2 ± 11.5	69.1 ± 12.4
VFQ‐25 sum score	76.8 ± 17.6	72.1 ± 14.0	73.6 ± 13.2	84.3 ± 11.7	59.2 ± 19.9	81.7 ± 13.3	88.4 ± 9.5	79.6 ± 17.5

sd = standard deviation.

* For some respondents, gender is missing.

### NEI‐VFQ‐25

The NEI‐VFQ‐25 is a vision‐specific QoL questionnaire consisting of a 25‐item base set of questions and a supplement of 14 additional items measuring vision‐related QoL. The NEI‐VFQ‐25 can be summarized into a ‘total component score’ and generates the following domains: global vision rating, near activities, distance activities, limitations in social functioning, role limitations due to vision, dependency, mental health, driving, peripheral vision, colour vision and ocular pain, ranged from 0 to 100. Most NEI‐VFQ‐25 items include five Likert scale answer categories. However, 19 items also include a sixth ‘opting out’ category, ‘Stopped doing this for other reasons or not interested in doing this’, which is treated as a missing value.

### Item selection

In a first selection, we excluded items with more than 10% missing values. This was a strict criterion, as for the proposed ‘routing procedure’ (see below), missing data would have been problematic. The previously described ‘opting out’ category caused all of the, due to missing values, excluded items. So some items could be dropped beforehand for contextual reasons, as they only applied to subgroups of patients: such as ‘car driving’ or ‘visiting movies, plays, or sports events’ and an opting out was present. The NEI‐VFQ‐25 has two pairs of items which could be seen as duplicates, namely item 1 and A1, and item 2 and A2. The main difference is that the second of both deviated in answer categories compared with all other items, and was therefore excluded.

**Figure 1:**
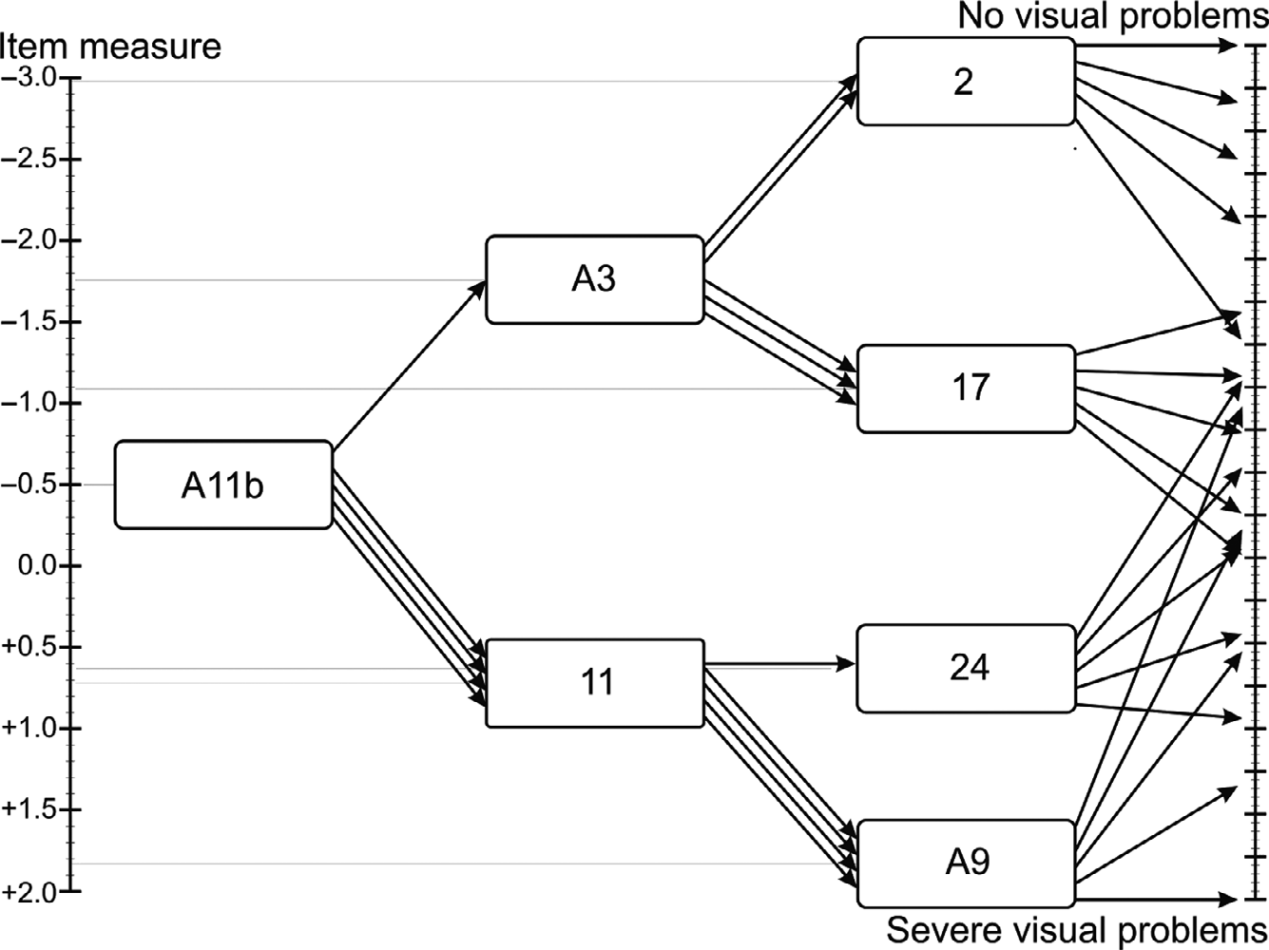
Schematic presentation of the VFQ‐3oo7. Rasch analysis allows expressing the respondents' performance on the same latent trait as the item measure. First item A11b, in the middle of the latent trait, is administered. Depending on the answer, the respondent is routed through the questionnaire. Every arrow represents an answer category, and the split is determined by the median of the item. In the end, only three out of the seven items could be used, where the answers navigate patients to a fitting trait level.

Following the recommendations made by Reeve et al. (2007), we first performed a confirmatory factor analysis (CFA). In case of a poor fit, a principal component analysis (PCA) was performed to check uni‐dimensionality by the following requirements: (i) the explained variance of the first component should be at least 40%, (ii) the first eigenvalue should be at least five times higher than the second one and (iii) items should load at least 0.50 on the first component. A Monte Carlo PCA for parallel analysis was performed as an additional evaluation of the eigenvalues (Watkins 2006). Cronbach's alpha was calculated for the remaining items, as well as the person separation and person reliability indices. These indices are considered better suited as a measure of reliability for Rasch analysis. The person separation index should be at least 2.0 and the person reliability at least 0.80 (Linacre 2019). In case the assumption of uni‐dimensionality was not sufficiently met, a second dimension would be analysed, and a second short form VFQ would be constructed for this dimension. All items complying with these unidimensional requirements were analysed with a generalized partial credit model (gPCM). This is a two parameter Rasch model for ordered categories. The gPCM assumes equal differences between the answer categories over the items. This makes an ordering of the items on the latent trait possible, based on the item measure, and provides item differentiation parameters. Rasch analysis also allows to express the respondent’s performance on this same latent trait, the person measure (Embretson & Reise 2013). The operationalization of our aim was to create the ‘VFQ 3 out of 7’ (VFQ‐3oo7), by reducing the NEI‐VFQ‐25 to seven items. Seven items were deemed sufficient for a broad classification in an computerized administration, where routing reduced the number of presented items to three. The selection was done by classifying the latent variable into seven classes, and from each class, the best discriminating item was selected for the final version of the VFQ‐3oo7 (Figure 1).

### Validating VFQ‐3oo7

To test the statistical validity of the VFQ‐3oo7, several analyses were performed.

#### Fit statistics

Infit and outfit measures are mean squares provided by Winsteps, to detect poorly fitted items. Mean squares greater than 1.0 indicated an underfit to the model, and mean squares less than 1.0 indicated an overfit, where values between 0.7 and 1.3 were considered acceptable (Wright et al. 1994).

#### Differential item functioning (DIF)

DIF may occur when a test item does not have the same relationship to a latent variable across two or more groups (Embretson & Reise 2013). That means that persons from different groups who have the same position on the latent trait will have a different outcome. In this study, DIF was discerned for the different eye disorders. For large samples, the DIF *t*‐value is unduly often significant(Tristan 2006). To compensate for this, we applied the normalizing procedure described at the Rasch Organization site, and adjusted the standard errors with √(N/100).

#### Multilevel structure

The patient samples included pre‐treatment baseline scores and one or more follow‐up measures after treatment. All these measures were included. This was not in accordance with the independence of measurement assumption. Generally, this can be overcome by performing multilevel analyses, the persons form the upper level, their repeated measures the lower level. Unfortunately, the IRT program we applied is not capable of performing multilevel analyses. Therefore, we applied a procedure to estimate the effect of neglecting the multilevel structure (Mallinson 2011). This procedure provides a visual presentation of the deviation caused by the dependency. Additionally, we preferred a formal test for the deviation and calculated the mean absolute difference (MAD), and compared this to the standard errors of the person measures. The MAD then should be within the 95% confidence interval of the gPCM person measures, thus lower than 1.96 times the standard error. This procedure is described in more detail in Appendix S1 and Fig. S1‐S5.

#### Precision

In order to determine the sensitivity level of the VFQ‐3oo7, we used the relative precision method (McHorney et al. 1992; Gothwal et al. 2009). Although Gothwal et al. (2009) applied *F*‐tests for the calculation of precision, we preferred random effect models as these make more efficient use of all data. We used the data of our largest longitudinal sample and calculated the relative precision with the t‐values, with the Likert‐score of the VFQ‐25 as reference. We excluded the items on car driving because these were excluded in the first place by Pesudovs et al. (2010), Kowalski et al. (2012) and Fukuhara et al. (2013) as well. The remaining 32 items were Likert scored and Rasch scored, just as the selection of the items made by Pesudovs, Kowalsky and Fukuhara.

#### Disorder‐specific analyses

For practical reasons and optimization of generalizability, one uniform VFQ‐3oo7 is preferred; however, we performed separate analyses for the individual disorders, leading to different versions of the VFQ‐3oo7. We applied sensitivity analyses within the various samples in order to decide whether it is worthwhile to have different versions for each particular eye disorder.

#### Routing of the VFQ‐3oo7

By using routing, the number of presented items was reduced to three, as items out of the range would not be presented to the patient. For instance, if the first item already indicated that the patient had severe visual problems, items about minor problems would not be presented. For administration, the first item to be filled in was in the middle of the latent trait, the second on a quarter or three quarters, depending on the answer on the first item. The routing was determined by the medians. The answer on the second item determined which of the remaining four items will be presented as the third item (Figure 1). To successfully perform routing, items with more than 10% missing values were excluded in an earlier stage, as missing data made routing problematic.

#### Item weights for calculating the VFQ‐3oo7 score

Lastly, in an iterative procedure, weights for the VFQ‐3oo7 score were determined by applying a maximum Pearson correlation with the gPCM measure as criterion. These weights were rescaled so that, after a logit transformation, the scores had a range from 0 to 100, consistent with the range of the NEI‐VFQ‐25.

Confirmatory factor analysis (CFA) was performed with STATA version 15.1 [StataCorp, College Station, Texas 77845 USA], and gPCM was performed with Winsteps version 4.1.0 [Linacre, J. M. (2018). Winsteps^®^ Rasch measurement computer program. Beaverton, Oregon: Winsteps.com]. All other analyses were performed with SPSS version 25 [IBM SPSS Statistics for Windows, Armonk, NY: IBM Corp.],

## Results

We excluded all items about car driving, because these items included a large number of missing values (Table 2). Item A7 ‘sports’ had 12.1% and item 14 ‘movies, plays’ had 10.7% missing values and were excluded. These missing values were a result of a sixth answer category ‘Stopped doing this for other reasons or not interested in doing this’. The additional items A1 and A2 were similar to the regular items 1 and 2, but had a divergent number of answer categories, namely ten instead of five, and hence were also excluded. This resulted in 32 items for principal component analysis (PCA).

**Table 2:** Original NEI‐VFQ‐25 + 14 items, PCA loadings and number of missing values.

Item	Text	PCA loading	Percent missing values
1	General health	0.392	0.8%
2	Present eyesight	0.677	1.6%
3	Worry about eyesight	0.593	0.2%
4	Pain or discomfort	0.285	0.2%
5	Difficulty reading ordinary print in newspapers	0.820	0.4%
6	Difficulty with work or hobbies	0.798	1.4%
7	Difficulty finding something on a crowded shelf	0.820	0.6%
8	Difficulty reading street signs or the names of stores	0.813	0.9%
9	Difficulty going down steps, stairs, or curbs in dim light or at night	0.755	1.3%
10	Difficulty noticing objects off to the side while walking along	0.742	1.0%
11	Difficulty seeing how people react to things you say	0.825	0.9%
12	Difficulty picking out and matching clothes	0.734	1.5%
13	Difficulty visiting people in their homes, at parties, or in restaurants	0.779	1.4%
14	Difficulty going out to see movies, plays, or sports events		10.7%
15	Are you currently driving, at least once in a while?		0.9%
15a	If no: have you never driven a car or have you given up driving?		66.3%*
15b	If you gave up driving: Was that mainly because of eyesight?		86.2%*
15c	If currently driving: difficulty driving during daytime in familiar places		35.4%*
16	Difficulty driving at night		37.2%*
16a	Difficulty driving in difficult conditions		36.4%*
17	Do you accomplish less than you would like because of your vision?	−0.792	0.5%
18	Limited in how long you can work or do other activities?	−0.774	1.0%
19	Pain or discomfort keeps you from doing what you’d like to be doing	−0.541	0.6%
20	Stay home most of the time because of eyesight	−0.722	0.3%
21	Frustrated a lot of the time because of eyesight	−0.793	0.5%
22	Much less control, because of eyesight	−0.840	0.4%
23	Because of eyesight, I must rely too much on what other people tell	−0.835	0.4%
24	I need a lot of help from others because of my eyesight	−0.854	0.6%
25	I worry about doing things that will embarrass myself or others	−0.752	0.6%
A1	How would you rate your overall health, on a 0‐10 scale?		1.9%
A2	How would you rate your eyesight now, on a 0‐10 scale?		1.2%
A3	Difficulty reading small print on a medicine bottle, or on legal forms	0.749	1.5%
A4	Difficulty figuring out whether bills you receive are accurate	0.839	2.4%
A5	Difficulty shaving, styling your hair, or putting on makeup	0.759	2.8%
A6	Difficulty recognizing people from across a room	0.802	1.6%
A7	Difficulty in active sports or other outdoor activities you enjoy		12.1%
A8	Difficulty seeing and enjoying programs on TV	0.814	1.6%
A9	Difficulty entertaining friends and family in your home	0.763	1.9%
A11a	Do you have more help from others because of your vision?	−0.812	1.4%
A11b	Limited in the kinds of things you can do because of your vision?	−0.861	1.4%
A12	I am often irritable because of my eyesight	−0.694	2.2%
A13	I don’t go out of my home alone, because of my eyesight	−0.682	2.3%

Shaded items are included in Rasch analysis.

PCA = principal component analysis.

* These high missing values are partly a result of the limited amount drivers among the respondents.

The confirmatory factor analysis (CFA) indicated an insufficient fit: CFI (0.844), TLI (0.833), RMSEA (0.097) and SRMR (0.051). Following the recommendations by Reeve et al., we then performed an exploratory factor analysis. The explained variance was 56.50% by the first component and 5.05% by the second component. The following eigenvalues for the first four components were as follows: 18.08; 1.62; 1.30 and 1.01, respectively. The Monte Carlo PCA parallel analysis suggested that the first eigenvalue should be at least 1.187, the second 1.164, the third 1.147 and the fourth 1.13. Thus, according to this criterion a second and third factor might be present. However, the solution with two components resulted in one pain item (item 4). The three component solution included the two items on pain only in the third component (items 4 and 19), where the second component was entirely recessive. For this reason, we continued with the one‐component solution, and the second and third components were not suitable. This one‐component solution yielded two items with a too low component loading (<0.50); item 1 on the general health state (0.39), and item 4 on pain and discomfort around the eyes (0.29, Table 2). Therefore, 30 items were selected for gPCM analyses. These 30 items had a person separation index of 3.79, a person reliability index of 0.93 and a Cronbach's alpha of 0.975.

Ordering of the items on the basis of the latent trait, classification and selection of the most discriminating items per class, resulted in the selection of the items 2, A3, 17, A11b, 24, 11 and A9 (Table 3).

**Table 3:** Items in GPCM model sorted by item measure and categorized in seven categories.

Item	Item measure	Discriminative value	Original domains	Pesudovs Two scale approach		Kowalski	Fukuhara
2	−2.95	1.00	General vision	V11†			†
3	−1.95	0.54	Mental				
A3	−1.75	0.95	Near	V15*, †			†
6	−1.09	1.13	Near	V12†		†	†
17	−1.08	1.18	Role		S12†	*, †	†
9	−0.95	0.89	Distance	V13†			
5	−0.91	1.07	Near	V14†			†
18	−0.74	1.01	Role		S10†	†	
A11b	−0.49	1.33	Role		S9*, †		*, †
8	−0.36	1.06	Distance	V10†			†
10	−0.32	0.87	Peripheral	V7*, †			
22	−0.29	1.11	Mental		S11†		*, †
21	−0.12	0.94	Mental				
A8	−0.04	1.11	Distance	V6*, †			†
A11a	0.12	1.09	Role		S4*, †		*, †
7	0.22	1.15	Near	V9†		*, †	
A4	0.26	1.13	Near	V4*, †			*, †
A12	0.29	0.72	Mental				
19	0.37	0.25	Pain				
23	0.56	1.11	Dependent		S8†		*, †
A6	0.61	1.06	Distance	V3*, †			†
24	0.63	1.18	Dependent		S6*, †		†
A5	0.66	0.93	Near	V2*, †			
11	0.72	1.16	Social		S3*, †	†	†
25	1.12	0.98	Mental		S7†	†	†
20	1.18	0.93	Dependent		S5†	†	*, †
13	1.24	1.11	Social		S1†		*, †
12	1.34	1.02	colour	V1*, †		*, †	
A9	1.80	1.17	Social				
A13	1.92	1.02	Dependent		S2*, †		

Shaded items have the highest discriminative value in a category and are included in the final selection for the VFQ‐3oo7. Pesudovs and Kowalski selected item 14, which we did not take into account because of >10% missing values. Pesudovs also selected A7, which had also >10% missings in our sample. Two scale approach of Pesudovs: V = Visual functioning and S = Socio‐emotional item. Both items were ordered by severity.

GPCM = generalized partial credit model.

* First selection by Pesudovs, Fukuhara and Kowalski.

^†^ Final selection by Pesudovs, Fukuhara and Kowalski.

### Fit statistics

The item infit mean square measure for the 30 item gPCM analysis was 1.08 and the outfit measure was 0.90. For the seven‐item analysis (including all scored categories), these measures were respectively 1.04 and 0.91. All these measures were well within the acceptable range of 0.70 and 1.30.

### Differential item functioning (DIF)

Item 2, which was the first item at the best seeing side of the latent variable, showed DIF for uveal melanoma patients (adjusted t‐value = 2.94; p = 0.003). For these patients, this item was ranked second, while this item was far on the well seeing side for the other six populations. Note that there were 49 DIF tests applied (seven items times seven eye disorders). A Bonferroni correction would result in a corrected significance level of p = 0.001.

### Multilevel structure

The mean absolute difference (MAD) for the total sample was 0.130, thus lower than 1.96 times the standard error (1.96*0.073 = 0.142). The MADs were larger for retinal detachment (0.161; CI < 0.403), glaucoma (0.172: CI < 0.214) and uveal melanoma (0.173; CI < 0.243), and smaller for corneal disease (0.101; CI < 0.292) and macular degeneration (0.100; CI < 0.212), but the same held for the standard errors. Within each patient group, the MADs were within acceptable confidence limits.

### Precision

We applied our data on the treatment of macular degeneration for the precision analyses, as this was our largest sample with at least two measurements. We had 336 baseline measures and 285 follow‐up measures. Rasch scoring performed better than Likert scoring for all selections (Table 4). The selection of 11 items by Fukuhara et al. (2013) yielded the largest relative precision, followed by the visual function scale of Pesudovs, the VFQ‐3oo7 and the selection of Kowalsky.

**Table 4:** Precision of various selections of the NEI‐VFQ‐25, based on our macular degeneration sample.

Scale	Method	Number of items	Macular degeneration	
			t‐value	Relative precision
VFQ‐32	Likert	32	−4.539	100.0
	Rasch	32	−5.366	118.2
VFQ‐3oo7*, *		3	−3.940	86.8
VFQ‐7*, †	Likert	7	−4.806	105.9
	Rasch	7	−4.919	108.4
Fukuhara	Likert	11	−4.927	108.6
	Rasch	11	−5.585	123.1
Kowalsky	Likert	6	−2.460	54.2
	Rasch	6	−3.284	72.4
Pesudovs‐visual function scale	Likert	6	−4.912	108.2
	Rasch	6	−5.098	112.3
Pesudovs‐socio‐emotional scale	Likert	7	−2.435	53.7
	Rasch	7	−2.894	63.8

* The VFQ‐3oo7 has a weighted sum score that is derived from Rasch analyses.

^†^ These are all the 7 items applied in the VFQ‐3oo7 without routing.

### Disorder‐specific analyses

Rasch analyses within the data of the different eye disorders generally led to other selections of items. In the retinal detachment sample, the solution yielded a correlation of 0.928, which was 0.005 higher than the general solution in this sample (*r* = 0.923). In all other samples, the sample‐specific solution led to a lower correlation than the overall solution.

### Routing of the VFQ‐3oo7

The first item presented to every patients was item A11b ‘Are you limited in the kinds of things you can do because of your vision’? as it is in the middle of the latent trait (Fig. 1). Answer category ‘a’ led to item A3, where categories ‘b’ to ‘e’ led to item 11. Categories ‘a’ and ‘b’ on item A3 led to item 2, and categories ‘c’ to ‘e’ led to item 17. Category ‘a’ on item 11 led to item 24 and categories ‘b’ tot ‘e’ led to item A9.

### Item weights for calculating the VFQ‐3oo7 score

**Figure 2:**
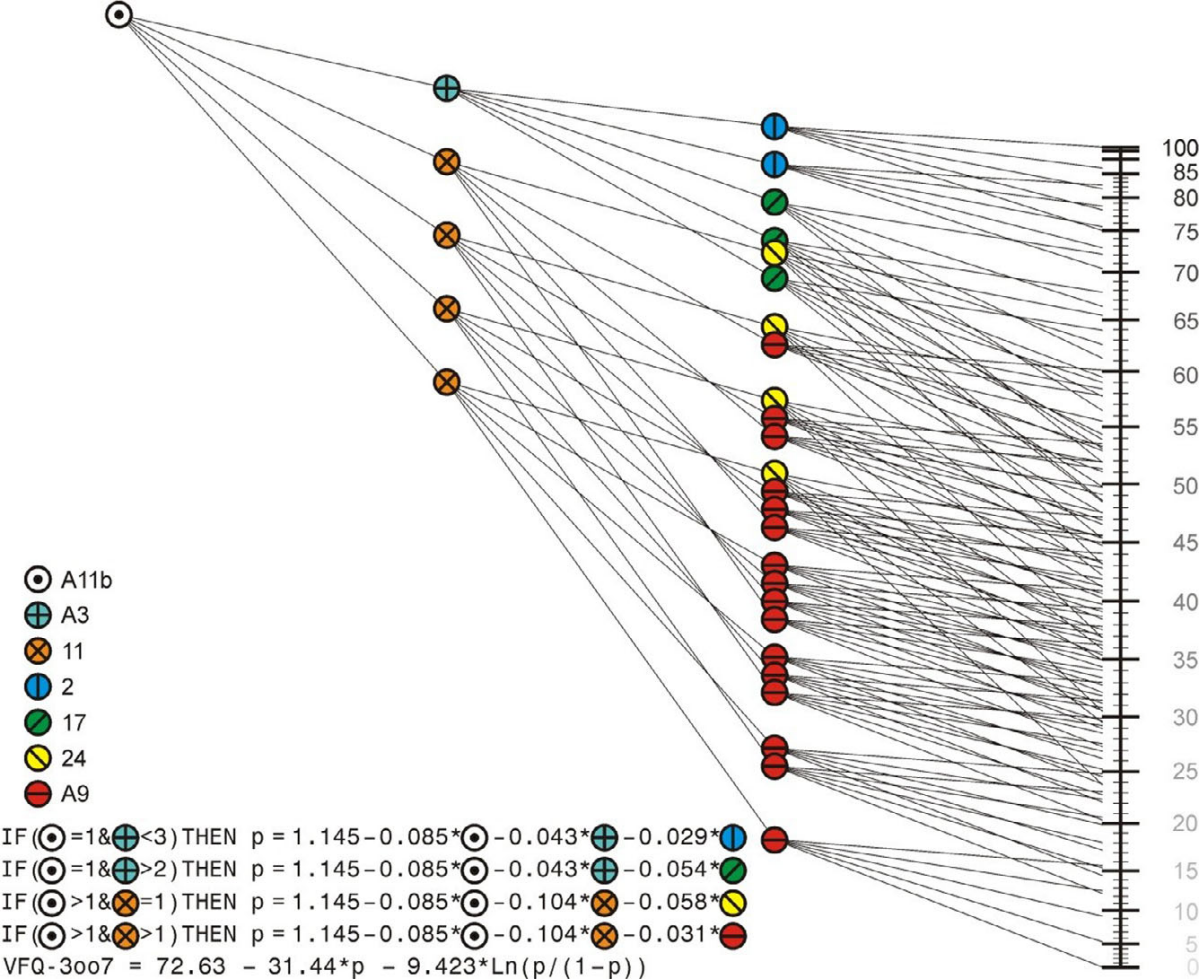
Visual presentation and calculation of the VFQ‐3oo7. The upper left symbol starts at 1.145. Then, 0.085 times the response on the first item (A11b) is subtracted. When this first response is 1, the next question is A3, and 0.043 times the response on A3 is subtracted. The same procedure holds for the third question. When the response on the first question is larger than 1, the second question is 11, and subsequently 0.104 times the response on this question is subtracted. Then, the third question follows in the same way. Note that the scale is not linear, but a logit scale. The routing is based on medians, therefore question A9 is most frequent.

The optimal weights gained from the iterative procedure are presented in Fig. 2. This solution resulted in a correlation of 0.924 with the person measures of the 30‐item gPCM.

## Discussion

### Principal findings

The main goal of this study, to reduce the number of items for routine use of PROMs in regular care, is particularly important for patients with eye disorders. First, problems with eyesight may cause more difficulty in answering questionnaires. Second, they represent mainly an elderly population, having a higher comorbidity. A higher comorbidity in turn demands more follow‐up visits and treatments, with more frequent PROM questionnaires as a consequence. Hence, these patients are probably more subject to respondent burden.

This study reduced the number of items of the NEI‐VFQ‐25, including additional items, from 39 to the predetermined three administered items out of seven. A one‐dimensional structure was considered fitting. During the item selection, five items were removed due to a large number of missing values and two items were removed as a result of similarity to other items. Two other items showed too low component loadings and were therefore excluded. The last step of reducing the remaining 30 items to seven items was accomplished by Rasch analysis. By routing, only three items out of the seven need to be administered. The VFQ‐3oo7 showed a small loss of information compared with the total score of the NEI‐VFQ‐25: correlation 0.927 and a relative precision of 0.868.

### Our principal findings in relation to the existing literature

Successfully shortened versions of the NEI‐VFQ‐25 have been produced earlier. However, our aims were more rigorous in reducing the number of items to as few as 3 out of 7; in addition, our methods differed from those used before. In concordance with Fukuhara et al. (Fukuhara et al. 2013), we also excluded the three driving items. They used a scree plot and eigenvalue criterion to distinguish the number of dimensions, which more or less matched our uni‐dimensionality criteria 2 and 3, namely the first eigenvalue should be at least five times higher than the second eigenvalue, and items should load at least 0.50 on the first component. For item selection, a more stringent criterion was applied than in previous studies (Pesudovs et al. 2010; Fukuhara et al. 2013), which used a loading of 0.40, with an explained variance of 0.40^2^ = 16%. A loading of 0.50, indicating an explained variance of 25%, will provide more reliable items.

Fukuhara et al. (2013) and Kowalski et al. (2012) intended to retain information on all domains, and therefore included at least one item on the domains general vision, near activities, distant activities, vision‐specific social functioning, vision‐specific mental health, vision‐specific role difficulties and vision‐specific dependency (Table 3). Our study did not aim to include every domain, but to retain the largest range as possible of the visual spectrum. The result is that we did not include items for the distant activity and mental health domains.

Pesudovs et al. (2010) discerned two scales: a visual functioning scale and a socio‐emotional scale. In our data, we did not find a socio‐emotional dimension. When we evaluated the two and three component analyses, these components only included the two items on pain (items 4 and 19).

The precision analyses showed that the selection of Fukuhara et al. (2013) distinguished best between the measurements in our data of patients with macular degeneration. It must be noted that they also used the most items. Fewer items obviously lead to a lower precision. The six items that are selected for Pesudovs' vision scale perform virtually as well as all 32 items. However, Pesudovs' social scale performs much less in this population. It may be that it better performs in other patient populations. Also, Kowalski's selection does not perform very well in this population. The VFQ‐3oo7 performs less than the selections of Fukuhara and Pesudovs. This imprecision is likely to be the price for the large reduction of items, if the relative precision would also be calculated relative to the number of items, the VFQ‐3oo7 would score best.

### Limitations

Item 2 ‘present sight’ showed marginally significant DIF in the uveal melanoma patients. Generally, the item at the best seeing end of the continuum was present sight. However, in the uveal melanoma population, item 3 ‘worry about eyesight’ was the item that was most sensitive at the best seeing end. Patients with uveal melanoma experience not so much visual problems but have other worries. This may be explained by the dooming and worrisome nature of uveal melanoma and carcinoma in general, as there is a significant risk of losing an eye or even worse, die. Apparently, and logically, item 2 of the VFQ‐3oo7 had a slightly different meaning for the uveal melanoma patients, as these patients experience less visual problems. This indicates that the VFQ‐3oo7 may be less sensitive for uveal melanoma patients with mild visual problems.

A inevitable consequence of our aim to reduce the VFQ‐25 to only three questions to be answered is that it excludes the possibility to cover all original subscales. For specific eye disorders, it can be appropriate to add one or a few relevant items considered indispensable, that is for glaucoma patients the item on pain can be added, in particular, because pain was hardly represented in the one‐component solution.

### Future perspectives

In this study, we focused on shortening an instrument to measure vision‐related health status. However, at least for some eye disorders, additional perceived outcome domains may be relevant. For example for glaucoma, the burden/side effects of treatment have been identified as a relevant outcome domain (Somner et al. 2012). For specific eye disorders, future studies should focus on identifying additional domains and items, while preserving the requirement of a very short instrument.

The latest development in Rasch includes computer adaptive testing (CAT) in the clinical application. This creates interesting possibilities, but one needs to employ a full CAT infrastructure. With the methodology presented here, such CAT infrastructure is not necessary and thus the VFQ3oo7 can be employed more easily. With the VFQ‐3oo7, ophthalmologists have a user‐friendly tool to monitor the patients' perspective on their visual functioning in regular care. The use of the VFQ‐3oo7 offers possibilities to explore the patients' perspective, without the cumbersome administration of the long original NEI‐VFQ‐25. In our hospital, the VFQ‐3oo7 is now implemented. Patients fill in the computer‐based questionnaire before each consultation. The outcome is directly presented in the electronic patient file and visible to the ophthalmologist. It is most appreciated in the clinical communication when clinical outcomes contradict with the outcome of the VFQ3oo7 or when the outcome of the VFQ‐3oo7 shows signs of improvement or deteriorations over time. It is up to the ophthalmologist to use the outcome of the vfq3oo7. This could aid the dialogue between ophthalmologists and patients, and could help to substantiate a referral to low vision specialists or psychologists.

## Conclusions

The goal of this study was to reduce the number of NEI‐VFQ‐25 items to seven items of which three are to be administered by the patient while retaining a high distinctive capacity, to make it suitable for routine PROM measurement in clinical practice. Correlating 0.927 with the criterion, the VFQ‐3oo7 has succeeded in realizing this goal. The VFQ‐3oo7 also appeared suitable for various eye disorders. The very short, but valid, VFQ‐3oo7 can be applied to evaluate the patient's perceived vision‐related health status in routine evaluations of treatments in regular care, with only little burden for patients.

## Supporting information


**Appendix S1.** Rasch analysis of repeated measures.

**Fig. S1.** Mean Rasch measures of the first 10 retinal detachment patients at baseline and follow‐up.
**Fig. S2.** Mean Rasch measures for the first 10 glaucoma patients at baseline and follow‐up.
**Fig. S3.** Mean Rasch measures for the first 10 patients with corneal diseases at baseline and follow‐up.
**Fig. S4.** Mean Rasch measures for the first 10 patients with macular degeneration at baseline and follow‐up.
**Fig. S5.** Mean Rasch measures for the first 10 patients with uveal melanoma at baseline and follow‐up.

## Data Availability

The VFQ 3oo7 can also be administered on paper, and scored by means of an Excel file that is digital available on request.
