# Supplementary material for: A very short version of the Visual Function Questionnaire (VFQ‐3oo7) for use as a routinely applied Patient‐Reported Outcome Measure
Source: Acta Ophthalmol. 2020 Mar 18;98(6):618–26. doi: 10.1111/aos.14378 (PMC7496098; doi:10.1111/aos.14378)
Supplement: Supplementary file 1 — Appendix S1. Rasch analysis of repeated measures. [file AOS-98-618-s001.docx]

**Appendix S1.**

## Rasch analysis of repeated measures.

The procedure described in https://www.rasch.org/rmt/rmt251b.htm is as follows:

"Repeated measures are common in rehabilitation studies where patients are scored on assessments at both admission and discharge. There are often intermediate or follow-up data collection periods in addition. The amount of change in patient functional status is an important indicator of rehabilitation quality. In order to determine that it is indeed the patients who have changed and not the item difficulty, constant "anchor" values are needed to fix item difficulties at admission and discharge (or any other time point) within a common frame of reference. Yet creating an anchor file is problematic.

One approach is to create a file of item anchor values by "stacking" the admission and discharge data so that each item corresponds to one column, and each time-point for each person is a row in the combined dataset. However this approach may violate the Rasch assumption of local independence in the observations because some characteristics of the patients span time-points. Yet creating item anchor values from either the admission data only, or the discharge data only, and then applying those values to the whole data set may not be reasonable either. Generally, patients are quite disabled at admission to rehabilitation so performance on difficult items of assessment tools are rarely observed or are scored in their lower rating-scale categories. At discharge, patients have often made considerable improvement and most will be scored in the top categories of easier items. At either admission or discharge, some items will be "off-target" compared to patient ability and, for some items (the hardest ones at admission, and the easiest ones at discharge), only one or two categories of the rating scale may be observed.

This suggests a different approach:

1) Create a random sample of patients across the time-points so that each patient is only in the data set once but all time-points are equally represented.

2) Analyze this "random" data set and estimate the item difficulties and Rasch-Andrich thresholds. Save these values in anchor files. They become the definitive set of item difficulties, defining the measurement framework of the latent variable.

3) Apply the anchor files to the estimation of the person abilities at all time points. This can be done either with each time-point in a separate dataset or with all time-points stacked in one dataset. There will be no interaction between the observations of each person at the different measures because they are isolated from each other by the item anchor values.

4) With all the data stacked, and a time-point code in each patient record, do an item-by-time-point DIF analysis to verify that nothing unexpected has happened to the items."

We then added the following step:

5) Run an analysis without anchors on all available cases, and compare the results.

The suggested approach was applied to our dataset including 640 patients who had at least two measures in time, 58 patients with retinal detachment, 81 with a corneal disease, 108 glaucoma, 282 with macular degeneration and 106 uveal melanoma patients. First (1), a random sample was selected across the 2 time points so that each person was only in the "random" dataset once but both time points were equally represented. Then (2), this random sample was used to create the anchor files. Finally (3), the anchor files were used in the estimation of the 640 patients with both Time 1 and Time 2 records. For comparison, an unanchored "stacked" analysis of all 1280 available records for all patients at both time-points was performed. In this last analysis, the estimates for Time 1 and Time 2 would be influenced by local dependency across time-points, if there is any.

The Figures show the relationship between the "stacked" and "anchored" measures of the first 10 persons at baseline and follow-up. We can see that for the retinal detachment and glaucoma patients (Figures A1 and A2) the dependent measures are lower in the low regions, see e.g. patients 3, 8 and 9. There is less difference for higher scores. For patients with carneal diseases and uveal melanoma patients virtually no differences are observed (Figures A3 and A4).

In deliberation with the author of the Winsteps program, Mike Linacre, we concluded that this procedure is not so much a solution to the dependency and multilevel problem, but a way to estimate a potential bias caused by it. Generally, the necessity to include an extra amalysis level can be determined with a deviance test (Singer & Willett 2003). When the deviance test is not significant, the multilevel solution does not differ from the single level analysis and the more parsimonious single level analysis is preferred.

The procedure described above provides a visual presentation of the deviation caused by the dependency. The authors of this paper preferred an additional formal test for the deviation and we calculated the mean absolute difference (MAD), and compared this to the standard errors of the person measures. The MAD then should be within the 95% confidence interval of the gPCM person measures, thus lower than 1.96 times the standard error. The MAD for the total sample is 0.135 logits, while the SEs of the stacked and anchored measures are both 0.074. Thus the MAD for the total sample is below the limit of 1.96*0.74=0.145, which is acceptable. As was to be expected from the figures, the MADs for retinal detachment (0.170), glaucoma (0.183) and uveal melanoma (0.184) were higher than for patients with corneal diseases (0.109) and macular degeneration (0.099). All MADs are within the 95% confidence limits, thus it can be concluded that a multilevel analysis is not necessary.

Table A1. Mean absolute differences and standard errors of the stacked and anchored Rasch measurements.

| Visual disease | MAD | Stacked | | Anchored | |
| --- | --- | --- | --- | --- | --- |
|  |  | SEM | [95% CI] | SEM | [95% CI] |
| Retinal detachment | 0.170 | 0.210 | 0.412 | 0.210 | 0.412 |
| Corneal diseases | 0.109 | 0.149 | 0.292 | 0.149 | 0.292 |
| Glaucoma | 0.183 | 0.113 | 0.221 | 0.113 | 0.222 |
| Macular degeneration | 0.099 | 0.110 | 0.216 | 0.110 | 0.216 |
| Uveal melanoma | 0.184 | 0.127 | 0.250 | 0.127 | 0.250 |
| Total | 0.135 | 0.074 | 0.146 | 0.074 | 0.145 |

MAD = mean absolute difference between stacked and anchored

SEM = standard error of the mean

[95% CI] absolute 95% confidence interval
