# Supplementary material for: A very short version of the Visual Function Questionnaire (VFQ‐3oo7) for use as a routinely applied Patient‐Reported Outcome Measure
Source: Acta Ophthalmol. 2020 Mar 18;98(6):618–26. doi: 10.1111/aos.14378 (PMC7496098; doi:10.1111/aos.14378)
Supplement: Supplementary file 2 — Fig. S1. Mean Rasch measures of the first 10 retinal detachment patients at baseline and follow‐up. Fig. S2. Mean Rasch measures for the first 10 glaucoma patients at baseline and follow‐up. Fig. S3. Mean Rasch measures for the first 10 patients with corneal diseases at baseline and follow‐up. Fig. S4. Mean Rasch measures for the first 10 patients with macular degeneration at baseline and follow‐up. Fig. S5. Mean Rasch measures for the first 10 patients with uveal melanoma at baseline and follow‐up. [file AOS-98-618-s002.docx]

# Figures


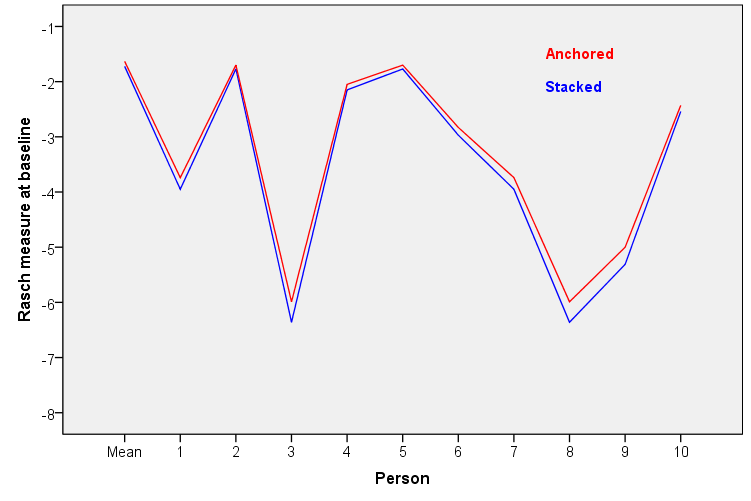

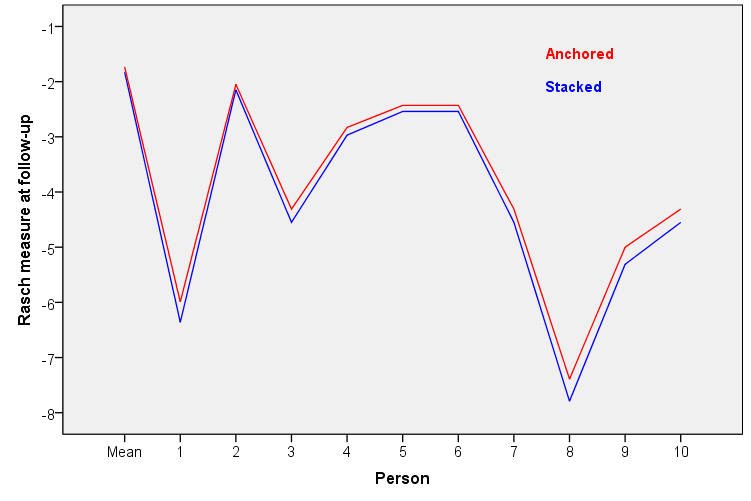
**Figure S1.** Mean Rasch measures of the first 10 retinal detachment patients at baseline and follow-up.


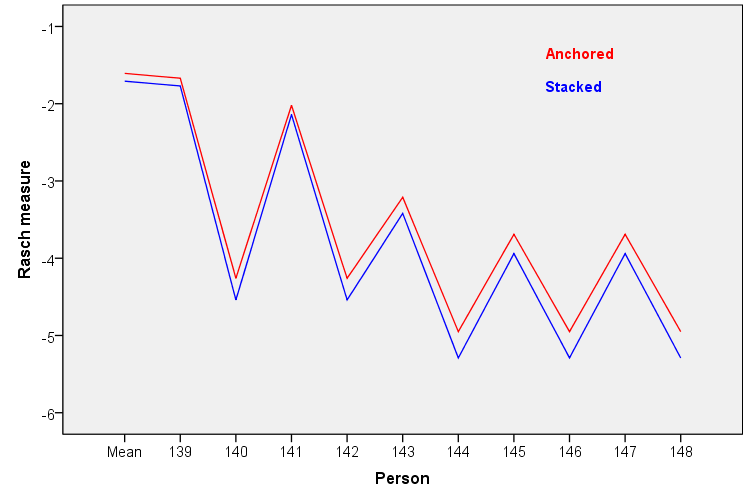


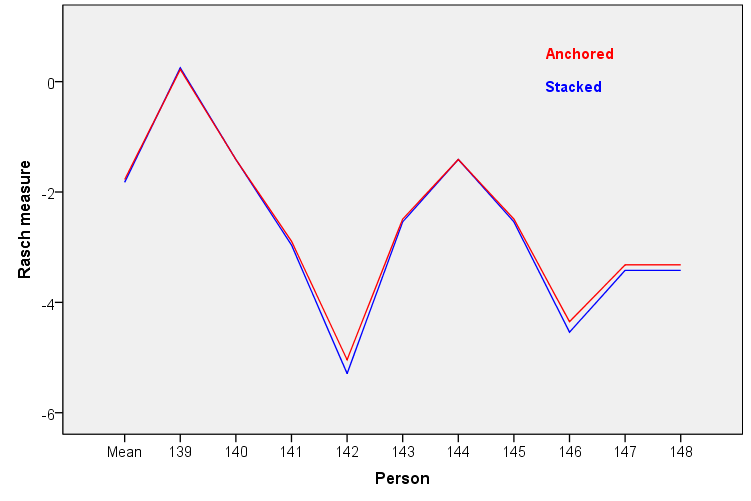


**Figure S2.** Mean Rasch measures for the first 10 glaucoma patients at baseline and follow-up.


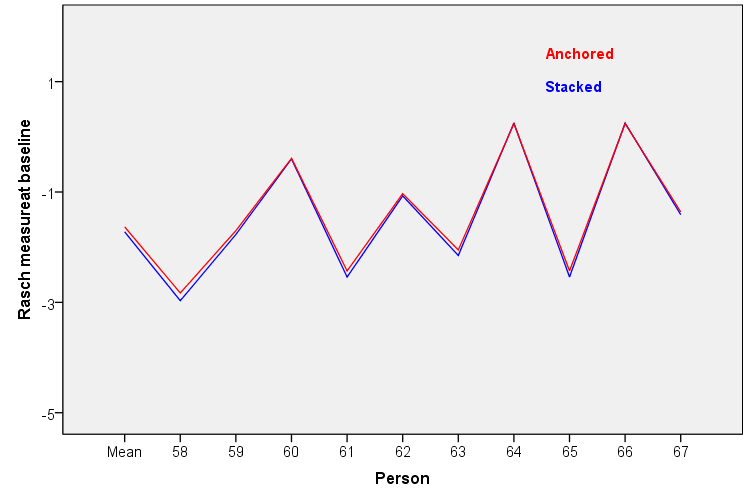

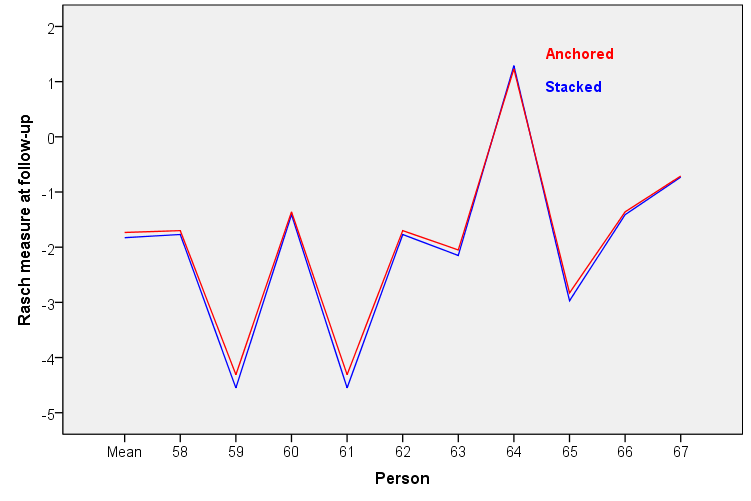
**Figure S3.** Mean Rasch measures for the first 10 patients with corneal diseases at baseline and follow-up.


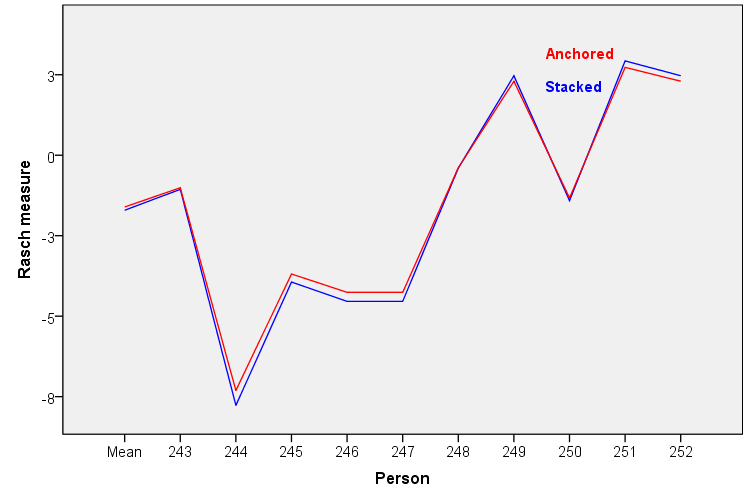

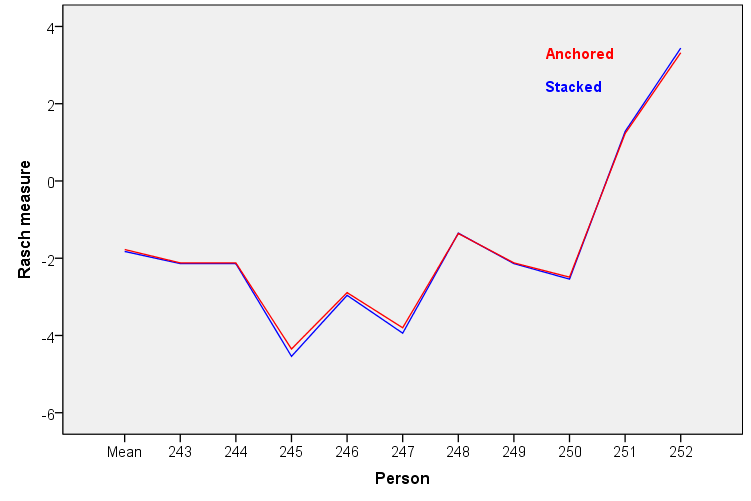


**Figure S4.** Mean Rasch measures for the first 10 patients with macular degeneration at baseline and follow-up.


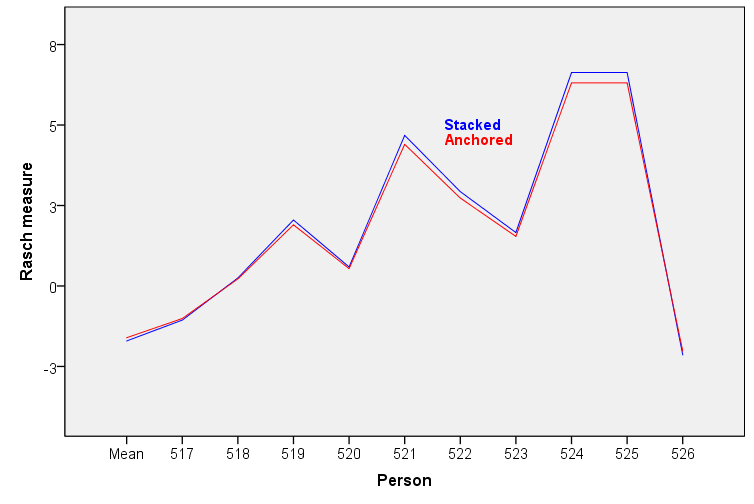

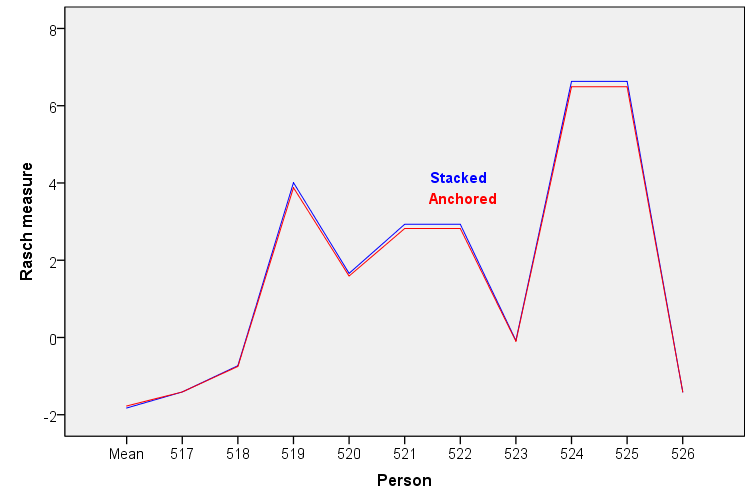
**Figure S5.** Mean Rasch measures for the first 10 patients with uveal melanoma at baseline and follow-up.
